# Crafting dental specialities in Iran: insights from a qualitative study

**DOI:** 10.1186/s12903-024-05332-0

**Published:** 2025-01-03

**Authors:** Tayebe Rojhanian, Michael Aryan Kya, Shahram Yazdani, Mohammad-Pooyan Jadidfard

**Affiliations:** 1 Department of Community Oral Health, Faculty of Dentistry, Hormozgan University of Medical Sciences, Bandar Abbas, Iran; 2 Student for Doctor of Dental Surgery, School of Dentistry, Medical University, Tehran, Iran; 3 Department of Medical Education, Virtual School of Medical Education and Management, Shahid Beheshti University of Medical Science, Tehran, Iran; 4 Departments of Community Oral Health, School of Dentistry, Shahid Beheshti University of Medical Sciences, Tehran, Iran

**Keywords:** Dental education, Advanced education, Health workforce, Stakeholders, Specialisation

## Abstract

**Background:**

Different countries have varying dental specialities, shaped by diverse factors. The determinants influencing the development of these specialities differ between developed and developing countries. This study aimed to explore the factors contributing to the establishment of dental specialities in Iran, a developing country with a wide range of recognised dental specialities.

**Methods:**

A qualitative case study was carried out, involving the review of 25 in-depth interviews and 47 documents. The data were organised using Atlas.ti (version 7.57) software and analysed through content analysis. This process included transcribing the interviews, identifying meaning units, abstracting content, categorising codes, and developing themes.

**Results:**

The results identified three key factors influencing the development of dental specialities in Iran: stakeholders, contextual factors, and the specialisation process. Stakeholders encompass influential figures such as abroad-trained specialists, the government, the Ministry of Health and Medical Education, and scientific associations, along with their position, perceptions, and power. Contextual factors include cultural norms, sociopolitical relationships, political shifts, economic conditions, and academic disciplines. The process of establishing new dental specialities revealed several gaps, including the absence of formal needs assessments, advocacy plans, career planning, effective partnerships, and adequate evaluation mechanisms.

**Conclusion:**

Contextual factors have played a crucial role in shaping dental specialisation in Iran, driving the formation of ideas in this field. Key players, including dentists trained abroad, have significantly influenced this process, often motivated by the desire to mirror practices in other countries. However, it did not address the specific oral health needs of the Iranian population. Due to limited awareness and the cost disparity between specialised and general services, there has been little public demand for dental specialisation. However, the process of establishing these specialities faces significant gaps that need to be addressed.

**Supplementary Information:**

The online version contains supplementary material available at 10.1186/s12903-024-05332-0.

## Introduction

Specialisation has brought numerous benefits across various fields [1] and is often regarded as a marker of scientific advancement [2]. It has also been crucial in modern medicine [3], significantly enhancing the quality of life [1].

Medical specialities first appeared in Paris in the late 1830s [3]. In the early nineteenth century, the medical profession consisted of three main groups: physicians, surgeons, and pharmacists [3]. Over time, dental services have been separated from medicine and surgery; dentists provided dental services as a separate group [4].

The study of dental specialities (DS) in different countries indicates a great variety [1, 5–9]. The differences in the number of specialities and specialists in different countries depend on the cultural structure of each country [10]. This variety shows an inequality in organisation of DS [11].

Specialities typically emerge from clinical, technological, and scientific advancements [12]. In addition, people’s growing expectations and demographic changes justify the need to develop specialised fields [5]. New specialities usually emerge outside the existing knowledge domain [13], and a set of required and relevant knowledge and skills is created for the specific field, which specialists acquire through experience and training [12, 14].

Organising new specialities as part of governance in the health sector is essential [15]. If the formation of new specialities is unplanned and uncontrollable, it can lead to contradictions in providing health services in developing and developed countries [16]; decisions about the number and expansion of specialities have challenged choosing between fairer healthcare systems that focus on primary services or increasing the number of specialists to improve service quality [17].

Medical specialities are increasingly being established in some countries [15]. Iran is among the countries with the highest number of recognised specialities in dentistry (10 specialised fields and two Ph.D. courses). The number of DS in Iran, a developing country, is comparable to the developed countries [18]. New specialised courses and dental fellowships are continuously proposed to be established in Iran. However, little information is available on how DS were established in Iran and what factors influenced its decision-making and idea formation. Factors affecting specialisation in developing and developed countries are different. In developed countries, the state has a more limited role in specialisation, unlike in developing countries the state plays a more significant role. Most available information is about developed countries [10].

Achieving the health system’s goals necessitates careful planning regarding workforce diversity [1] and numbers. Establishing a new speciality is a significant intervention at the health system level, with implications for the entire spectrum of service provision. Therefore, awareness of the factors influencing decision-making for establishing DS in Iran, a case of developing countries, is essential because it will be evidence of how such decisions were made in these countries. Investigating this issue can identify potential strengths and weaknesses in the decision-making processes of establishing DS in developing countries. Such information can also help provide recommendations for improving conditions. This study aims to investigate the reasons and factors affecting the formation of DS in Iran.

## Methods

To design and analyse this study, we employed qualitative research methods. Given that specialisation is a complex social phenomenon, the case study focused on investigating the emergence of DS in Iran.

### Data collection

The study gathered data from 25 interviews and a review of 47 documents. The study group comprised experienced individuals in Iranian dentistry, including past and present officials of the Dental and Specialised Education Council. We used purposive and snowball sampling to select participants. Data collection took place from September 2019 to February 2020. Five interviews were excluded—one due to the interviewee’s advanced age, another due to incomplete information, and three due to insufficient responses—resulting in 25 interviews with 22 respondents (Table 1).

**Table 1:** Number and grouping of participants in interviews

Number of respondents	Organisational grouping
9	Senior Administrators
8	Educational Administrators
5	Senior Professionals in Dentistry
22	Total

Before conducting the interviews, we explained the research objectives to the participants, either via telephone or in person. We assured them that all ethical standards for conducting a qualitative study were upheld [19]. Initially, participants were asked about their awareness of the factors influencing the establishment of DS and what those factors were. To refine our approach, three pilot interviews were conducted, after which the topics covered were reviewed to identify potential gaps. Based on these reviews, additional questions were developed. We then developed an interview guide to facilitate semi-structured interviews, specifically designed for this study, and the interviews conducted have not been previously published. Subsequently, two more pilot interviews were conducted, during which further questions were incorporated based on emerging themes. The interview questions were open-ended to allow participants to express their views freely and explore the subject in depth (The interview questions are included in the Supplementary File 1).

The interviews were conducted in a semi-structured format to ensure consistency while providing flexibility to probe further into emerging themes. Written informed consent was obtained from the interviewees either during the briefing session or at the start of the interview. The interviews, conducted face-to-face by TR, were audio-recorded. Participants were encouraged to share their experiences and perspectives on the creation of DS in Iran. Each interview lasted approximately between 35 and 50 min. The interviews took place in participants’ offices, ensuring convenience and comfort for the participants. Following each session, a complete verbatim transcript was prepared and provided to the interviewee for review, allowing them to verify the accuracy of the data and suggest any necessary corrections. Data collection continued until theoretical saturation was reached. To maintain confidentiality, a code was assigned to each interviewee, ensuring their anonymity regardless of organisational affiliation, and it was used throughout the analysis.

The documents used in this study were selected through consultations with interviewees, searches on the Ministry of Health’s website, and research into the history of dentistry in Iran. A collection of available documents, including meeting minutes of the Dental and Specialised Education Council (33), training programmes of specialised dental courses (12), articles, and books on the history of dentistry in Iran (2) were reviewed. The documents were analysed to make use of the evidence available and validate the result of the interviews. A total of 47 documents, articles, and books were reviewed.

### Analysis

The data were analysed using Atlas.ti software (version 7.57) [20]. Content analysis method was used for data analysis [21].

Since TR conducted and transcribed the interviews, she was deeply familiar with all the discussions. After preparing precise, verbatim transcripts of each interview, the transcripts and related documents were thoroughly reviewed. Meaning units were identified, and initial codes were extracted from these units. The coding process was developed using both deductive and inductive approaches. The content of each interview was segmented into meaning units based on their definitions, and research codes were generated by abstracting the relevant meaning units. Subcategories were then formed, and themes were extracted by assessing the patterns among the existing codes. To ensure the validity and reliability of the data, methods such as peer debriefing, member-checking, and triangulation were employed [22]. Peer debriefing involved consulting with colleagues who were not directly involved in data collection to critique the findings. Member checking was performed by sharing the findings with participants to validate the interpretations. Triangulation was achieved by comparing the interview data with document data to ensure consistency. The study adhered to ethical guidelines, including obtaining written informed consent, ensuring confidentiality, avoiding bias, providing participants the right to withdraw at any time, and maintaining their anonymity.

Subsequently, the themes related to the key actors involved in the establishment of DS in Iran were independently evaluated by two members, SY and TR. They assigned a score to each actor, indicating their level of influence on a scale of 1 to 4 (with 1 representing the lowest influence and 4 the highest). In cases of disagreement between the coders, consensus was reached through a review of the interviewees’ quotes.

## Results

**Fig. 1:**
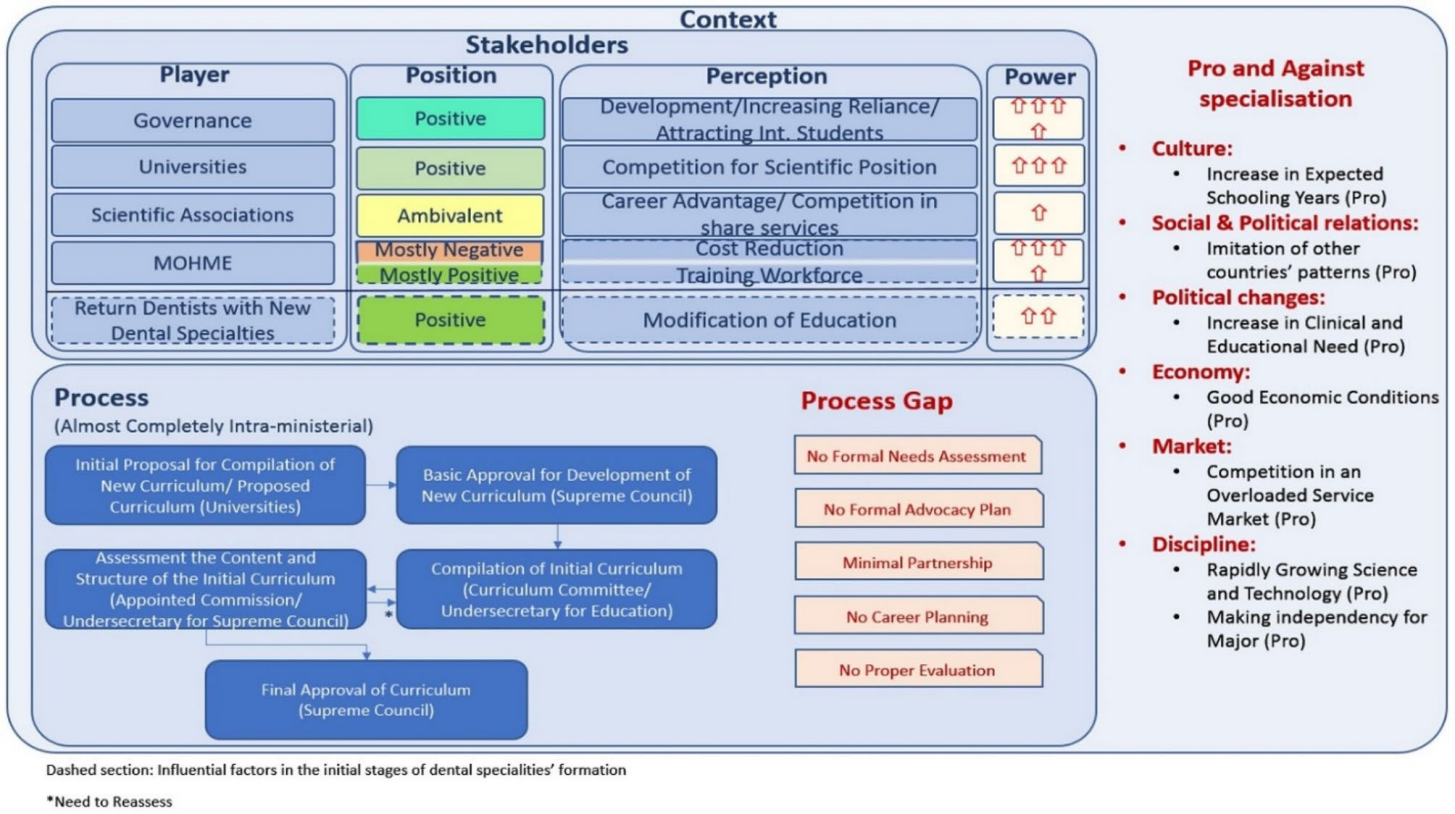
Factors contributing to the making of dental specialities in Iran

After data analysis, three main themes were extracted including: (A) Stakeholders: This theme encompasses the various players involved in the establishment of dental specialities, their perceptions, positions, and power. (B) Process and process gap: This theme highlights the procedural aspects of establishing dental specialities and identifies gaps in the process. (C) Context: This theme addresses the contextual factors, including those that support and hinder the establishment of dental specialities (Fig. 1).

### Stakeholders, player

#### Return of abroad-trained dentists with new DS

According to the interviewees in this study, individuals who were awarded scholarships to study abroad were the initial catalysts for the interest in DS in Iran. Most of these students, having received government-funded specialised education, were dedicated to returning and contributing their expertise to the country.

*“The cornerstone of dental development was laid by these people educated abroad. Some capable people got scholarships and used the country’s wealth and that those people returned and developed the country”* (P.13, Senior Administrators, Male).

As reported by some interviewees, the acceptance of foreign-educated specialists in both executive and scientific roles played a crucial role in advancing DS in Iran. Their knowledge of international laws and regulations, coupled with their practical experience, offered valuable insights into the progression of dental specialisation in the country. Additionally, their education at prestigious global institutions bolstered their professional credibility, facilitating the acceptance and implementation of their recommendations.

“*Some of these [individuals] did the administrative work themselves. For example*,* I was the vice president of XX and YY… Well*,* with such positions*,* I could help develop my field*.” (P.7, Senior Administrators, Male).

#### Universities

Some participants emphasised the pivotal role of Iranian universities in the expansion of DS. The establishment of the ‘Educational Planning Committee at the University and Specialised Levels’ and the ‘Specialised Education Council and Postgraduate Studies of the Specialised Council’ within each faculty has been instrumental in the development of DS.

“*Specialised education was part of the demands of the universities when this happened.*” (P.9, Senior Professionals, Female).

#### Ministry of health and medical education

According to some participants and official documents, the initiation of DS was overseen by the Ministry of Health and Medical Education (MOHME), in collaboration with the “Secretariat of the Council for Dental and Specialised Education” and the “Secretariat of the Supreme Council for Medical Science Planning”. The Secretariat of the Supreme Council for Medical Science Planning serves as the highest authority for policy formulation and macro-level educational planning in medical sciences.

*“The Secretariat of the Dental and Specialised Education Council is responsible for assessing the educational needs of dental disciplines*,* specialised and support groups*,* evaluating and determining the content of educational programmes and methods*,* assessing and determining the needs of human resources in dental fields*,* and determining the criteria for specialist training in dental schools”* (Document. 1).

#### Government

Insights gathered from interviewees indicate that significant efforts were made before the revolution to enhance Iran’s scientific standing. During that period, the government’s support and approach were crucial in approving the establishment of DS within the MOHME. Obtaining government approval was essential to secure adequate funding for restructuring human resources within the healthcare system. One of the interviewees commented:

*We needed the government’s approval to get enough money to have specialities. Without satisfaction of politicians and giving us money*,* we can’t do it.*

#### Scientific associations

Some interviewees suggested that the establishment of specialised groups and scientific associations has played a vital role in advancing DS in Iran.

“*Graduates of some specialisations abroad formed the specialised association of their field*,* so before that*,* we did not have specialised training in this field in the country*,* and the associations were effective in it.”* (P.4, Educational Administrators, Female).

However, some interviewees highlighted existing opposition to the formation of DS, primarily from medical speciality associations.

“*Dr. XX was very involved with the medical disciplines to establish this field because they kept saying that this is our right. There was a lot of resistance to the establishment of this field by the medical group*,* and medical XX [specialists] resisted creating this field.”* (P.21, Senior Professionals, Male).

#### People

Some interviewees claimed that there was no demand from the public at the beginning of dental specialisation. Two main reasons have been mentioned for why people did not feel the need for specialised dental services: lack of grassroots organisations and awareness about specialised services. Many individuals considered certain oral conditions, such as age-related tooth loss, as normal and did not seek treatment. Access to general dental services was limited, let alone specialised treatments.

“*People had nothing to do with these* things *and were living hand-to-mouth. They were struggling for their basic needs. People did not feel the need for specialisation before its advent*” (P.9, Senior Professionals, Female).

### Stakeholders, perception

#### Modification of the education

Based on the accounts provided by interviewees, upon the return of dentists trained abroad with new DS, they were employed in government positions to offset their education expenses. Upon their return, scholarship holders made changes based on their observations abroad, including simulating the educational systems of the countries they had studied in.

“*Abroad-trained specialists improved the educational system in Iran by implementing the same system that they experienced overseas*,* including establishing specialities*” (P.10, Educational Administrators, Male).

#### Competition for scientific position

As recounted by interviewees, universities aimed to have more DS to match those of the top universities worldwide.

“*They wanted to be equal to the best universities in the world and be like them*” (P.9, Senior Professionals, Female).

#### Career advantages/ competition for share services

Some interviewees claimed that the professional benefits associated with specialisation were identified as the primary drivers for advocating for the establishment of specialities.

“*Becoming a specialist has many benefits for dentists*,* and these benefits were effective in supporting the idea of developing specialities”* (P.1, Senior Professionals, Male).

Some interviewees stated that medical speciality associations opposed the formation of DS that overlapped with some areas of their speciality.

“*Every few years*,* a big fight happens between X [medical specialists] and Y [dental specialists] because these disciplines overlap and for economic reasons”* (P.3, Educational Administrators, Male).

#### Development/ attracting international students/ increasing reliance

The general aim of dental speciality education in Iran was explained as follows: “*The educational curriculum aims to train [specialists] who have achieved the standards of knowledge*,* beliefs and opinions*,* and practical skills up to national and global standards. In addition*,* the graduates should provide high-quality preventive and therapeutic services*,* provide educational services*,* and have an active role in expanding the boundaries of knowledge and research within the framework of their speciality field*” (D. 35–37, 39, 40, 42–44).

According to the 20-year vision document of the Islamic Republic of Iran, attaining a leading position in advanced science and knowledge in Southwest Asia and the ability to produce science and technology are outlined as goals until 2025. Various DS have been proposed to achieve these goals by imitating successful models from other countries. Establishing these specialities in Iran aims to provide high-quality general and specialised dental education and attracts predominantly international students from Islamic countries.

“*One of the ways for a country to progress in the field of knowledge is to have different specialists who do better research* with *the knowledge they have in the field of science and cause the development of science and technology and cause the country to be known*,* in particular*,* they make us scientifically prominent among the countries in the region*,* and this is a privilege for the country to attract foreign students*” (P.20, Educational Administrators, Female).

Findings from participant interviews highlight that governments aimed to gain public support by fostering development and innovation. They supported proposals that involved creating new DS and academic fields. One respondent commented:

*There was a national determination for the progress of the country*,* especially in the scientific fields of the country*,* that’s why the specialities were formed because the governments wanted people to see that the country is progressing and to support them.*

#### Training workforce

According to the testimony of interviewees, the shortage of dentists was the reason for the formation of DS. To address this shortage, dental specialists were needed to provide training and education for general dentists. Additionally, the provision of specialised dental services was another factor that contributed to the formation of DS.

“*At some point in the past*,* we did not have dentists. Dentists and doctors used to come from Bangladesh and abroad. They did not even understand our language*” (P.7, Senior Administrators, Male).

*“When you are going to have dentistry major*,* you will have some needs*,* and the most important requirement is the teacher*,* who should be a dental specialists”* (P.2, Senior Administrators, Male).

#### Cost reduction

According to some interviewees, MOHME was instrumental in creating DS initially, but today, the high expenses due to popularity of specialisation among dentists and dental students have raised concerns. To mitigate these challenges, solutions are needed.

“*The Ministry of Health had a positive approach to specialities previously*,* but now they do not know how to control it. In our country*,* when something and a field is formed*,* it is difficult to reduce the number or remove the field*.” (P.6, Educational Administrators, Male).

### Process and process gaps

The process of DS establishment is showed in Fig. 1.

Based on feedback received from interviewees, the specialisation of dentistry in Iran was carried out without conducting a needs assessment, and the reason was the lack of knowledge about the concept in the early years of specialisation in the country. In the beginning, the process of specialisation development was not commensurate with the oral health needs of the country. One participant mentioned:

*I think there was no scientific information about needs assessment at all at that time*.

According to many interviewees, no proper evaluation has been performed for dental specialisation in Iran. Some of the interviewees identified the lack of career planning, formal advocacy plans, and minimal partnerships among the stakeholders in the process of forming DS. Several participants stated that sometimes, decisions were made before the results of the evaluations and consultation were announced, and the policy evaluation has not been done. One respondent said:

“*I have many memories of going to visit*,* and before I could present the outcome of my visit*,* [the related] decision had already been made*”.

### Power

In Iran, the formation of dental specialities is primarily influenced by governance and the MOHME, with universities also playing a significant role. Ultimately, foreign-educated dental specialists and dental associations have less power and influence compared to other groups in this field.

### Contextual factors (pro specialisation)

#### Cultural factors

According to interviewees, higher education and dental specialisation are prevalent trends in Iran. Improving social and financial status are among the motivating factors for pursuing specialised education. Additionally, the growing demand for specialised dental services among patients contributes to dentists’ increased interest in specialised education.

‟*There is a lot of dental specialists in our country*,* that is*,* it is fashionable to specialise and everyone wants to be a specialist*.” (P. 8, Senior Administrators, Male).

#### Social and political relations

In accordance with what interviewees disclosed, the most crucial factor in making DS in Iran was political relations with United States and Britain, which led to emulation. Scientific relations with developed countries were an integral part of the development process that the government underwent during those years. Therefore, with the expansion of relations with Britain and the United States, dental specialisation has occurred in American and sometimes British protocols. One participant stated:

“*Another factor in creating specialisations was communication with the world especially the countries with which our political and social relations were better*,* such as America and England*,* based on which planning was done*”.

#### Political changes

According to the interviewees, the revolution in Iran influenced the establishment of specialised fields. The revolution, in particular, resulted in a surge in demand for specialised dental education due to the emigration of numerous experts following the regime change.

“*Regarding the revolution … there was a break*,* and then there was a cultural revolution*,* and all universities were closed for two years due to the cultural revolution … And it caused internal settlements in universities under the title of Islamisation of universities and a large number of professors were fired for various reasons*,* all in all*,* it naturally caused a decline in the education system*” (P.13, Senior Administrators, Male).

Some interviewees pointed out that during the Iraq-Iran war, the process of dental specialisation in the country did not change, and it was attributed mainly to the increase in treatment needs that arose after the war.

“*I think the war helped a little in the development of this field*,* because after the war*,* the traumas of the jaw and face increased and the need for this field increased.*” (P.10, Educational Administrators, Male).

#### Economic situation

Based on the interviewees, economic situation of country was one of the influential factors on specialisation of dentistry in Iran. It has been directly affected the allocation of scholarships needed for scientific development. During the establishment of DS, the country was in an ideal economic situation. The high prices of oil and other resources have been influential in the development process, including dental specialisation.

“*In the 1350s (Iranian calendar)*,* oil prices became more expensive and*,* naturally*,* the key to any development in the country is economic issues and undoubtedly*,* it is one of the aspects of economic development. So*,* they started sending some graduates abroad. Well*,* if the economic situation was not good*,* they certainly could not do it. Thus*,* it was definitely effective*” (P.11, Senior Administrators, Male).

#### Discipline

Some interviewees stated that advances in science and technology have been among the factors affecting specialisation. The spread of science, technology, and associated educational content, the impossibility of teaching specialised disciplines in general courses, and the emergence of some technologies have been practical on specialisation in dentistry. The trade of new technologies has also been influential in creating some specialities.

“*Many times it has been technology that has brought knowledge and occasionally technologies have been transferred into the dentistry from other fields … The world is moving towards technology*,* and educational companies have started training to sell their goods”* (P.15, Educational Administrators, Male).

According to the interviewees, the reasons for creating DS were the desire for independence, competition in attracting resources for dentistry, and a comparable identity with medicine. Despite the efforts to establish some specialised fields, there were few applicants; thus, it was tried to increase the desire for these fields by defining capabilities for its specialists.

“*We cannot sit and watch that the doctors have all the facilities and say we do not want anything … If we want to have a dental identity … We wanted to identify this field … some fields were not very popular and did not have a clinical aspect … some came to define capabilities in those fields to increase their attractiveness*” (P.14, Senior Administrators, Male).

Several interviewees mentioned that part of the competition for creating DS was related to disciplines that initially had a joint department. Separation of these fields made it possible to allocate resources and obtain faculty admission licences; therefore, it was performed. One respondent stated:

“*They wanted independence … their separation would have brought about independence*,* and they would be given facility*,* and they would be given the right to hire faculty*,* and they would increase their capacities*,* which is why they became three groups*”.

## Discussion

While many factors have been identified regarding the emergence and evolution of medicine [1, 10, 17, 23], particularly in developed contexts, there is limited research on DS, especially in developing countries. This study examines the key factors influencing the development of DS in Iran as a developing country.

This study highlights the crucial impact of specific contextual factors- such as international political relationships, social, and economic conditions- on the formation of DS in Iran. Since these factors often influence policy-making [24]; identifying them helps clarify their role in shaping specialisation as a policy. Döhler also has emphasised the critical role of contextual factors in the formation of medical specialities, noting that their impact varies across different countries [25].

For instance, political and social ties with Western countries [26–28], such as the United Kingdom and the United States, along with the imitation of their development models by returning graduates [26, 29–32], were influential factors in the emergence of DS in Iran. In this context, political and social relationships, along with changes in political and economic situations, are essential subsets of these contextual factors that have also been effective factors in the formation of DS in Iran. Additionally, several studies have highlighted the influence of political conditions and economic circumstances on decisions regarding medical specialisation in various countries [4, 10, 16, 33]. Overall, these contextual factors demonstrate a complex interplay that significantly affects the specialisation.

In addition to these contextual influences, development of science and knowledge has also been crucial in shaping DS in Iran. In this regard, several studies have identified science and knowledge development as important drivers of specialisation [13, 16, 17]. Our study found that higher education and dental specialisation are increasingly popular in Iran, with specialisation seen as a valuable career path.

Furthermore, factors such as higher income and social status have been identified as motivations for pursuing speciality training in Iran [34]. Research has also indicated that social status is a significant predictor of obtaining a speciality degree [10, 35]. Economic incentives, particularly income prospects, are important factors that influence both the decision to pursue specialisation and the choice of speciality [36, 37].

Notably, studies show that in some countries, specialisation has been pursued by following the models of more advanced nations to compete for scientific positions and attract international students [4]. In the case of Iran, the development of DS been notably influenced by these goals.

Furthermore, the need to train a skilled workforce to meet both educational and clinical demands emerged as significant factor driving the establishment of DS. However, in Iran, the absence of systematic need assessment and evaluation-driven policy-making has led to a situation where dental specialisation has been primarily driven by a desire to imitate developed countries, rather than by a genuine understanding of local needs. This situation has hindered the ability to correctly identify and address the underlying problems, resulting in issues that are not fully recognised and leading to misaligned priorities in this policy agenda. Overall, this misalignment underscores the importance of aligning specialisation efforts with local needs to ensure a more effective healthcare system.

In this study, the role of various stakeholders in the formation of dental specialities in Iran was demonstrated. Previous research has shown that stakeholders play a critical role in shaping healthcare policy, including the creation of specialised services [10]. Furthermore, effective health policy formulation requires the participation of various stakeholders in its design and implementation [38].

One of the key players identified in the formation of DS in Iran was the scientific associations. A research has also highlighted the influence of professional associations in driving the development of specialised healthcare services [10]. There is evidence to suggest that scientific and intellectual interests of certain groups can drive the emergence of specialities at key moments [17].

Another key player was the group of abroad-trained dental specialists, whose executive and scientific credentials played a major role in the making of DS. These specialists, through their exposure to advanced education systems, contributed valuable knowledge that influenced the local formation of DS.

However, the formation of DS was not without opposition. The emergence of DS introduced that potentially threatened the interest of some groups. Since these opponents were actors in DS policy-making, it is recommended that these opposing stakeholders, should be engaged in the evaluation and refinement of programmes to ensure that their concerns are addressed constructively. This approach is essential because opposition voices can significantly shape the outcome of healthcare policy [39].

Dental specialisation in Iran was primarily driven by a top-down approach, with directives from government authorities, including the MOHME, leading the implementation of policies [38]. Notably, there were no public demands for dental specialisation prior to the establishment of DS in Iran, partly due to the limited awareness of dental care needs among the general population. Typically, people usually request to make health policies when the burden and prevalence of the disease are high, crating pressure on policymakers. Despite the high prevalence of oral diseases in Iran [40] and the continuous increase in caries [41], the cost differential between specialised and general dental services [42] may have contributed to the lower demand for specialised services. This suggests that simpler services may better address the actual healthcare needs of Iranians. Moreover, studies have demonstrated that delivering cost-effective interventions at lower levels of care can improve access and equity, benefiting a large proportion of the population [1, 43]. Therefore, it is crucial to align dental specialisation policies with the actual healthcare needs of the population to create a more effective and equitable healthcare system.

This study aimed to identify factors influencing dental specialisation in Iran. One of the limitations of this study was the potential uncertainty regarding the accuracy of the information provided by interviewees. The study relied on a review of relevant documents and publicly available information on dental specialisation in Iran. However, it is possible that some relevant documents and information were not accessible to the researchers. To address these limitations, data were interpreted with caution, and triangulation, member-checking, and peer-debriefing were used to verify data. Given that this study has highlighted the significant role of stakeholders in the issue related to oral health, further research is recommended to explore their various stakeholder associated with this field. Additionally, as a detailed examination of the challenges in forming DS in Iran was not within the scope of this study, it is recommended that independent research be conducted to investigate these challenges and identify potential resolutions.

## Conclusions

This article highlights the factors influencing the formation of dental specialisation in Iran. Contextual factors have played a crucial role in shaping DS, contributing to the generation of relevant ideas. Key factors also include the influence of various stakeholders and their levels of power. Foreign-educated dentists were pivotal in introducing and adapting specialised education from other countries. The primary decision-makers in the establishment of dental specialities in Iran were the government and the Ministry of Health and Medical Education (MOHME). Additionally, there was limited public demand for DS due to a lack of awareness about these services and the cost disparity between specialised and general dental care.

The process of DS in Iran encounters several gaps, including deficiencies in needs assessment, advocacy planning, partnership development, effective evaluation, and career planning. To address these issues, it is crucial to focus on revising and improving the process. Engaging opponents can be a valuable approach for evaluating and critically assessing the specialisation process.

## Electronic Supplementary Material

Below is the link to the electronic supplementary material.

Supplementary Material 1

## Data Availability

The datasets used and/or analysed during the current study are available from the corresponding author on reasonable request.

## Abbreviations

DS: Dental specialities
MOHME: Ministry of Health and Medical Education
