# Supplementary material for: Crafting dental specialities in Iran: insights from a qualitative study
Source: BMC Oral Health. 2025 Jan 3;25:15. doi: 10.1186/s12903-024-05332-0 (PMC11697950; doi:10.1186/s12903-024-05332-0)
Supplement: Supplementary file 1 — Supplementary Material 1 [file 12903_2024_5332_MOESM1_ESM.docx]

**Examples of interview questions employed in the study**

| - Who were the key figures in the formation of dental specialisation in Iran, and what roles did they play? - Which organisations, groups, or individuals were involved in the creation of DS? - Describe the contribution of each influential group in shaping DS. - What role did individuals play in the specialisation of dentistry? - What were the motivations behind establishing DS? - What factors significantly influenced the process of dental specialisation in the country? - How did the process of specialisation in dentistry unfold? - What were the application procedures for confirming the establishment of specialities? |
| --- |
